# Durable Icephobic and Superhydrophobic Silicon Nanowire Surfaces

**DOI:** 10.1021/acsami.5c13616

**Published:** 2025-11-17

**Authors:** Seyed Mehran Mirmohammadi, Miika Heikkilä, Laura Fieber, Mohammad Awashra, Sara Hamed, Suprit Bhusare, Gaurav Mohanty, Robin H. A. Ras, Ville Jokinen, Sami Franssila

**Affiliations:** † Department of Chemistry and Materials Science, Micronova Nanofabrication Centre, 174277 Aalto University, Espoo 02150, Finland; ‡ Department of Applied Physics, Aalto University, Espoo 02150, Finland; § Materials Science and Environmental Engineering, Faculty of Engineering and Natural Sciences, 528748 Tampere University, Tampere 33014, Finland; ∥ Centre of Excellence in Life-Inspired Hybrid Materials (LIBER), Aalto University, Espoo 02150, Finland

**Keywords:** superhydrophobicity, metal-assisted chemical etching, hard coating, impact test, indentation, air plastron

## Abstract

Superhydrophobic surfaces hold great promise for various engineering applications. However, their fragility and limited durability in real-world scenarios pose significant challenges. Here, a durable superhydrophobic and icephobic surface is fabricated using the metal-assisted chemical etching method to create silicon nanowires within inverted pyramidal microstructures. Mechanical robustness is introduced by applying a hard coating to the structure through titanium film deposition, followed by annealing in a nitrogen atmosphere, which forms titanium silicide and titanium nitride. The hard-coated surfaces can endure up to 60 g of sand abrasion or 20 icing–shearing cycles while still retaining their superhydrophobic properties (advancing and receding contact angles of approximately 150°) and icephobic properties (ice adhesion strength of approximately 10 kPa).

## Graphical abstract

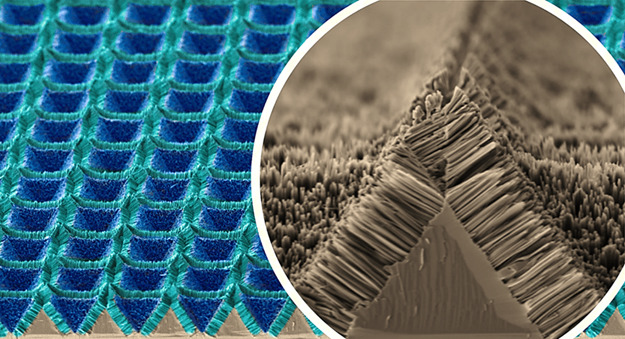

## 1 Introduction

A superhydrophobic (SHB) surface is characterized by contact angles (CAs) greater than 150°, sliding angles (SAs) less than 10°, and small contact angle hysteresis (CAH).^1,2^ Superhydrophobicity is inspired by nature and its fundamental mechanism involving both surface topography/rough structures^3^ and surface chemistry.^4^ Low surface energy materials, e.g., fluoropolymers^5^ and fluorinated silanes,^6^ have been extensively used on structured silicon or metal surfaces, resulting in excellent superhydrophobicity.

Silicon nanowires (SiNWs) with high-aspect-ratio structures play a distinct role in many applications, e.g., X-ray imaging,^7^ antibiofouling,^8^ optical sensors,^9^ gas sensing,^10^ and solar cells.^11^ A significant amount of research has been devoted to the design and fabrication of SiNWs through various approaches. Zhang et al.^12^ utilized a deep reactive ion etching method, employing a modified Bosch etching process and a gold nanoparticle-based mask, to obtain nanowires (NWs) with an average height of approximately 5 μm. Puglisi et al.^13^ fabricated SiNWs with diameters smaller than 5 nm through the vapor–liquid–solid method in the chemical vapor deposition plasma-based system. Irrera et al.^14^ reported a dense array of SiNWs with a length of about 2.5 μm, where a thin layer of gold was deposited on a silicon substrate, followed by a silicon etching step, where gold particles acted as a cathode. This method is known as metal-assisted chemical etching (MaCE), which is cost-effective, simple, scalable, and accessible even without cleanroom facilities. Various silicon nanostructures, including SiNWs, can be fabricated by MaCE through the control of etch rates, porosity, and etch profiles. For instance, the NWs’ length and shape can be controlled by the silicon substrate (resistivity, doping type), the catalyst (type, porosity), and the ratio of hydrogen peroxide (H_2_O_2_) to hydrofluoric acid (HF).^15^ In MaCE, a noble metal, e.g., gold,^16,17^ silver,^18^ and platinum,^19^ acts as a catalyst to reduce H_2_O_2_ in the etchant solution, generating electron holes (h^+^) through oxygen reduction. The holes are injected into the silicon substrate that is in contact with the noble metal, oxidizing the silicon, which is then dissolved by HF, resulting in the formation of NWs.^20^

To improve robustness and resist the Cassie-to-Wenzel transition, advanced surface topography has been developed. Research indicates that densely packed nanoscale structures, like nanowires, increase the Laplace pressure and resist this state transition.^21^ A downside of NWs is that they are fragile under mechanical loads due to their small contact area and high aspect ratio, resulting in high contact pressure. Nanoscale topography can experience critical damage from external forces, decreasing its mechanical durability and, consequently, its surface water-repellency properties. For example, a sandstorm can potentially damage exposed NWs. Therefore, in applications requiring extreme surface wettability, it is crucial to enhance the NWs’ mechanical robustness in order to achieve long-term stability of water repellency.^22^ Hsiao et al.^23^ showed that NW-based surfaces lost their water-repellent properties significantly, as the CA decreased to 114° after abrasion using 200-grit sandpaper due to the destruction of nanosized structures. In contrast, hierarchical structures exhibited excellent abrasion resistance, maintaining a CA of 155°. Another study reported that the CAH of MaCE-fabricated SiNWs on T-shape micropillars increased from 2° to 25° after abrasion by cloth wipes.^24^ Wang et al.^25^ achieved a mechanically durable SHB surface by utilizing interconnected inverted pyramids, which is known as an armored SHB surface. It was demonstrated that these inverted pyramidal structures can resist the highest loading during microindentation testing and thus show superior mechanical durability compared to pillars and pyramidal microstructures. This indicates that inverted pyramids, as microstructures, are protecting the fragile nanoparticles within the pyramids.

An effective anti-icing surface should prevent ice nucleation, slow down frost formation, and ensure that any ice that does form can be easily removed without damage. SHB surfaces can achieve low ice adhesion because of their low solid–water contact area, maintained by the trapped air plastron within the surface micro- and nanostructures, which keeps water in the Cassie–Baxter state during freezing. However, these surfaces have faced challenges, particularly in humid environments where frost tends to accumulate, and their overall durability has been problematic.^26^ Ice adhesion strength is typically determined by dividing the applied force by the ice area. Tensile shearing tests are commonly conducted using custom pull-off devices for this measurement.^27^ If the air plastron is lost and the Wenzel state is the wetting state, ice forms between surface features, and its adhesion strength significantly increases due to mechanical interlocking and a considerably greater solid water contact area. Therefore, when designing an icephobic surface, it is crucial to maintain the presence of the trapped air plastron at the solid–water interface to repel ice.^28^ Many application scenarios require the submersion of SHB underwater (e.g., antibiofouling).^29^ Maintaining air plastron stability underwater strongly indicates the durability of superhydrophobicity.^30^ A stable plastron can be achieved by a high degree of roughness, such as in hierarchical structures. However, mechanical robustness is also crucial to maintain the structure’s roughness degree and, thus, its ability to trap air.

In this work, we present the durable icephobic and superhydrophobic properties of silicon nanowires. The inherent fragility of the nanowires is effectively protected by the integration of shielding with microstructures and applying innovative hard coatings. The surfaces were fabricated using lithography and silicon wet etching to achieve inverted micropyramids, followed by the MaCE technique to obtain embedded NWs within the inverted pyramidal microstructures. Metal nitrides are recognized for their exceptional mechanical properties, making them an ideal choice for wear-resistant coatings. Thus, to enhance the mechanical robustness of the NWs, a titanium coating is applied, followed by annealing in a nitrogen atmosphere to form titanium silicide (TiSi) and titanium nitride (TiN). Our findings demonstrate that this hard coating significantly enhances mechanical robustness, reduces ice adhesion, and prolongs plastron lifetimes, likely due to preserving the nanoscale topography that ensures high Laplace pressure and kinetic energy barrier, thereby maintaining the Cassie state, even when subjected to mechanical stress.

## 2 Experimental Methods

### 2.1 Sample Preparation

#### 2.1.1 Photolithography and Silicon Wet Etching

A 4-in. silicon wafer (p-type, <100>, Siegert Wafer, Aachen, Germany) with a 500 nm thermally grown oxide was used as the substrate. Initially, the wafers were placed into a prime oven for hexamethyl disilazane (HMDS) treatment at 150 °C for 30 min, followed by spin coating with a layer of AZ5214E photoresist (MicroChemicals GmbH, Ulm, Germany) at a speed of 4000 rpm for 30 s, and soft bake on a hot plate for 2 min at 90 °C. Next, UV exposure for 3 s (hard contact), followed by development for 1 min in AZ 351B (AZ 351B: H_2_O = 1.8:9, Merck). Next, the oxide was etched in a buffered hydrofluoric acid (49% HF: 40% NH_4_F = 1:9) tank for 5 min at 32 °C, followed by the photoresist stripping in three steps: in acetone for 10 min ultrasonically, then acetone tank for another 1 min, and finally isopropanol (IPA) for 1 min, and then rinse with deionized water (DIW) tank for 3 min. The structured silicon wafers were then etched in 20% potassium hydroxide (KOH) solution at 80 °C for 20 min to achieve the inverted pyramids. Finally, the oxide mask was removed in 10% HF for 10 min at room temperature.

#### 2.1.2 Metal-Assisted Chemical Etching Method

The creation of NWs started with the sputtering of a 10 nm layer of gold (Figure S1). The MaCE process was carried out in an aqueous solution composed of 10% HF, 30% H_2_O_2_, and IPA in a 5:1:1 ratio for 30 min at room temperature, resulting in NW structures (Figure S2a). Then, samples were placed in a mixture of HCl and HNO_3_ (3:1) at room temperature to remove the residual gold.

#### 2.1.3 Hard Coating

A physical vapor deposition (PVD) system (model 09340, Angstrom) was used to deposit 90 nm of titanium through radio frequency (RF) magnetron sputtering. The chamber pressure was 9 × 10^–7^ Torr with an argon gas flow rate of 20 SCCM (standard cubic centimeters per minute). The deposition rate was 0.45 Å s^–1^. Following deposition, the titanium-coated samples were annealed in a vacuum furnace (ATV PEO-604) at 800 °C for 30 min in either an argon or a nitrogen atmosphere to achieve hard-coated surfaces (Figure S2b). The heating and cooling steps conducted under a vacuum of 28 mbar are summarized in Table S1, Supporting Information

#### 2.1.4 Silanization

First, the samples underwent oxygen plasma treatment (Tepla 400) for 1 min at 60 W power with a gas flow rate of 500 mL min^–1^ to introduce hydroxyl groups on the surface. Subsequently, gas-phase silanization using the self-assembled monolayers (SAM) method was applied to the hydroxyl-terminated surfaces. The samples were placed in a glass Petri dish, then a few milligrams of 1*H*,1*H*,2*H*,2*H*-perfluorododecyltrichlorosilane (GlpBio) were added to a small container inside the Petri dish. The Petri dish was capped and heated for 90 min at 100 °C.

### 2.2 Characterization

#### 2.2.1 Surface Characterization

Scanning electron microscopy (SEM, EBL Zeiss Supra 40) was employed to analyze the surface morphology. Energy dispersive spectroscopy (EDS, Zeiss Sigma VP) was performed to study the elemental analysis of hard-coated silicon nanowires after silanization, using an accelerating voltage of 12 kV. The CAs, both advancing and receding, were measured using a contact angle goniometer (THETA, Biolin Scientific) with the sessile droplet method. Advancing CAs were recorded from 2 to 5 μL droplet size, while receding CAs were measured from 5 to 0 μL, with a droplet pumping rate of 0.1 μL s^–1^. The fitting algorithm is polynomial. We plotted the CAs as a function of the baseline and checked the angle at the point where the baseline starts to advance or recede. SAs were determined using an in-house built goniometer with 10 μL water droplets at a tilting speed of 0.2° s^–1^. DIW was utilized for all the tests. Measurements were taken at three different locations on each sample, and the results are presented as the mean ± standard deviation.

#### 2.2.2 X-ray Diffraction Analysis

The crystalline structures of the samples were measured using X-ray diffraction (XRD, PANalytical X’Pert Pro-MPD, Cu Kα1 radiation); the Bragg angle was tested over a range of 25° to 55°. The lattice parameters were obtained by fitting the XRD pattern using HighScore software.

#### 2.2.3 Nanoindentation Tests

The indentation tests were conducted using an in situ Alemnis nanoindenter equipped with a sample-side load cell with a maximum load capacity of 0.5 N and piezo-actuated displacement range of 40 μm during indents. The load cell resolution was 4 μN, while the displacement resolution was <1 nm. The indenter setup had a compliance of 4.3 nm/mN. All experiments were performed in displacement-controlled mode with displacement targets. We used a Berkovich pyramidal indenter, whose tip area function was calibrated using continuous stiffness measurement (CSM) method on fused silica.^31^ This calibration ensured accurate determination of mechanical properties at shallow indentation depths.

#### 2.2.4 Sand Impact Test

The setup consists of a funnel holding a specific amount of sand, ranging from 1 to 60 g (Biltema, Sweden; size distribution: 200 μm - 1 mm, product number: 175401), placed above the test sample, which is inclined at 45°. The sand is released from the funnel, falling from a height of 30 cm at a rate of 8.5 g s^–1^ to impact the surface of the sample. The conditions correspond to an impinging energy of approximately 5 × 10^–7^ J per grain.

#### 2.2.5 Linear Abrasion Test

The abrasion tests were carried out using the Elcometer 5750 Taber linear abrader (Figure S3). Samples underwent abrasion for varying cycle numbers: 30, 50, and 100 cycles. A 3.5 N load was applied, with a stroke length of 12.7 mm, and the speed was set at 25 cycles per minute. Silicon carbide paper (P4000 and P2000, Mirka Ecowet) with grain sizes of 5 and 7 μm, respectively, was utilized for these tests.

#### 2.2.6 Chemical Stability Tests

Various solutions, including 6 M HCl, 6 M NaOH, and pure acetone, were prepared to evaluate the chemical stability of the hard-coated surfaces. The samples were immersed in these solutions for 6 h at room temperature, then rinsed with DIW and dried prior to conducting CA measurements.

#### 2.2.7 Ice Adhesion Test

The ice adhesion pull-off test used in this study closely resembles the system utilized in our prior research.^32^ The test was conducted using an Instron 4204 tensile tester equipped with a 100 N load cell (Figure S4a,b). The shear stress needed to detach the ice block was recorded and calculated by dividing the force by the ice surface area, with a pulling speed of 0.5 mm s^–1^. The test was performed at room temperature, 50% relative humidity, and included up to 20 cycles. The test ended when the ice adhesion strength values reached a plateau. Cylindrical Teflon molds with a 1 cm inner diameter, 2.2 cm outer diameter, and 2 cm height (Figure S4c) were used to make the ice blocks. The molds were filled with 1 mL of DIW and frozen for 3 h at −20 ± 1 °C with a relative humidity of 65 ± 2%.

#### 2.2.8 Plastron Stability Test

The experimental setup used in this investigation closely mirrors the system employed in our previous research.^33^ Briefly, a 2 × 2 cm sample of each prepared SHB surface was completely submerged in water. The dissipation of the plastron was monitored at controlled room temperature (21 ± 1 °C) using a video recording system (Canon 7D coupled with Canon Macro lens EF-S 60 mm −5184 × 3456 pixels) and cold light illumination (Godox LED126). The illumination was directed toward the SHB surface at a fixed position and angle. The surface is immersed in the water beaker with a water height of 4 cm. The beaker was then capped to prevent evaporation. The setup was enclosed within an isolated environment inside a black box to maintain consistent experimental conditions. The reflected light from the water–air interface of the plastron exhibited a bright, mirror-like appearance, indicative of the Cassie–Baxter state. The plastron coverage was recorded at 10 min intervals over a duration of 85 h. This procedure was applied to all four studied surfaces. Regarding plastron image analysis, all images captured during each experiment (510 images) were analyzed using ImageJ software. Plastron coverage measurements were conducted on a 4 cm^2^ area of each surface within all the images. The plastron coverage ratio for each image was calculated by dividing the plastron coverage by the surface analyzed area. A moving average was applied to the data set to smooth out the curves and reduce fluctuations. For a longer-term (months) observation of the plastron, a mirror-like appearance was observed by eye and captured by a phone camera (Figure S5).

## 3 Results and Discussion

### 3.1 Fabrication of Inverted Pyramidal Microstructures and Formation of NWs

Figure 1a illustrates the fabrication process for inverted pyramidal microstructures (i–v), NWs formation (vi–viii), and hard coating (ix–x). The width of the inverted pyramids was defined by the photomask design, while the ridge width could be adjusted by varying the etching duration in KOH. We chose 30 min of etching to decrease the ridge width to 1.2 μm (Figure S6a,b). Figure 1b,c shows the top view of bare inverted pyramids and SiNWs within the inverted pyramidal structures after the MaCE process, respectively. Cross-sectional views of both bare inverted pyramidal structures and SiNWs are shown in Figure 1d,e, along with more detailed views at increased magnification (Figure 1f,g). Silicon is primarily etched anisotropically using wet alkaline solutions, such as KOH or tetramethylammonium hydroxide (TMAH). When silicon is etched with these solutions, the etching rate is significantly slower for Si(111) planes compared to Si(100) planes because the (111) planes have a higher atomic density and stronger bonds, making them more resistant to chemical attack. Consequently, this anisotropic etching of silicon can create features such as V-grooves or pyramid-like structures.^34^ Wang et al.^25^ utilized TMAH (25% in water) to achieve inverted pyramidal structures. Similarly, they demonstrated that the width of the ridge could be controlled by adjusting the etching duration. For instance, in a structure with a 60 μm pitch, they achieved varying ridge widths ranging from 2.45 to 0.96 μm by modifying the etching time.

**Figure 1:**
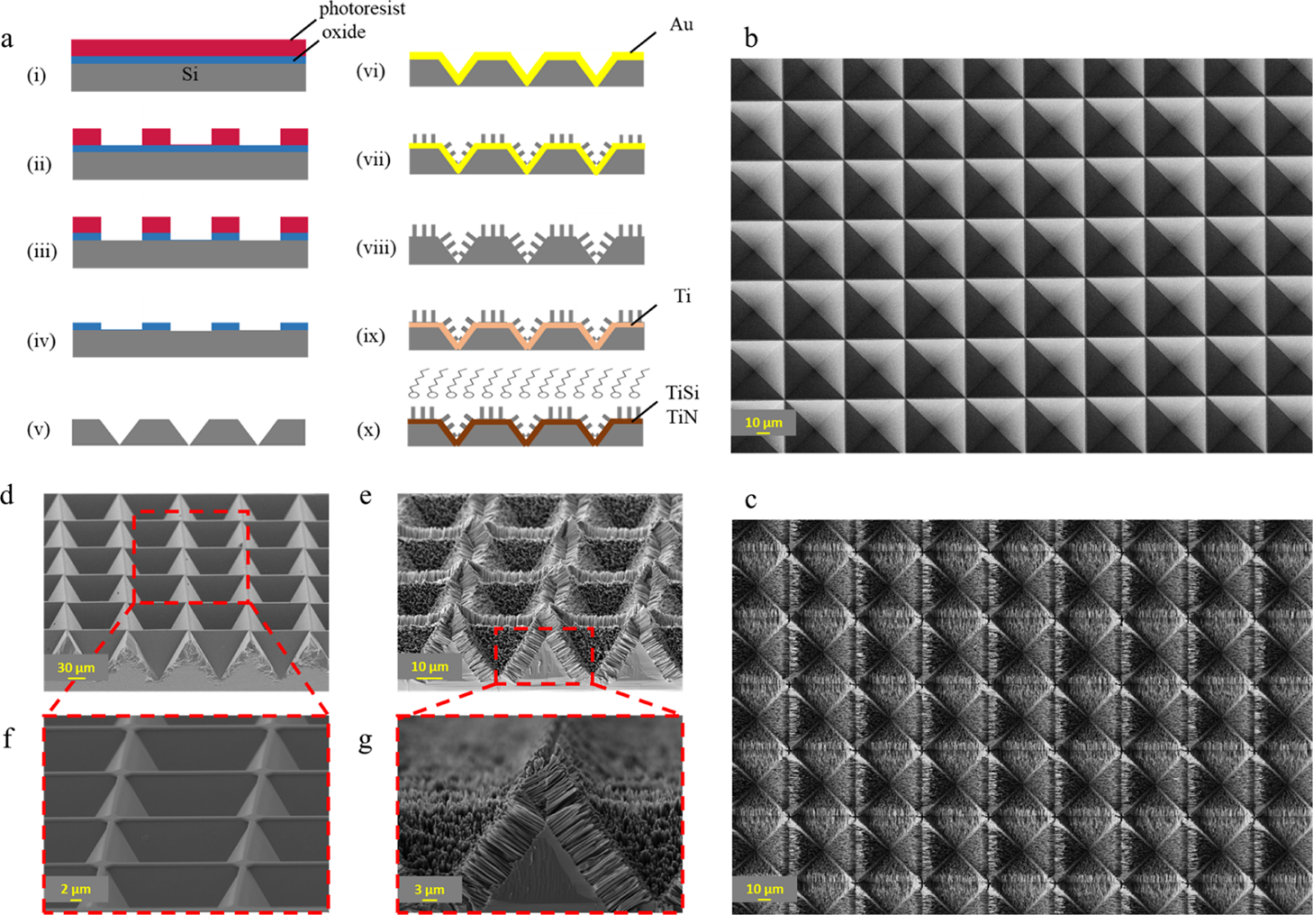
Fabrication process and SEM characterization of the surfaces. (a) Schematic illustration of the fabrication process: (i) photoresist spin coating, (ii) UV exposure and development, (iii) buffered HF oxide etching, (iv) photoresist stripping, (v) silicon KOH wet etching and oxide mask removal, (vi) gold deposition, (vii) MaCE, (viii) gold removal, (ix) titanium deposition, (x) annealing to form TiSi and TiN and silanization. Top-view SEM images of (b) bare inverted pyramids, and (c) SiNWs within the inverted pyramidal structures after the MaCE process. Cross-section views of (d) bare inverted pyramids, and (e) SiNWs. A closer view at higher magnification, displaying (f) the inverted pyramids and (g) the SiNWs within the pyramidal structures.

To assess the influence of IPA on the MaCE process, some samples were etched in an etchant solution that excluded IPA, both with and without stirring during the etching process. The findings revealed that different morphologies could be achieved. Without stirring during etching, NWs are not formed uniformly (Figure S7a). We noticed several large bubbles adhering to the surface within just a few minutes of etching, visible to the naked eye. Conversely, when stirring was employed during the MaCE process, the density of the NWs increased, but their growth was limited, with lengths reaching approximately 1–2 μm after 30 min of etching (Figure S7b). It has been reported that IPA is particularly effective as it reduces the surface tension of the etchant solution when mixed with HF, resulting in smaller gas bubbles. Our results align with those of Romano et al.^35^ who demonstrated that adding IPA as a surfactant to HF: H_2_O_2_ water solution enhances the uniformity and control of hydrogen gas release, enabling the creation of high-aspect-ratio silicon NW structures. On the other hand, Li and Duan^36^ applied extra energy by sonication to remove hydrogen bubbles during the etching process.

Figure 2 shows the length and density of the NWs, which could be adjusted by varying the MaCE duration. A small number of NWs were formed after 10 min of etching with a length of about 3 μm (Figure 2a). As the etching time increased to 20 min, more wires were grown, and the length increased to 5 μm (Figure 2b). After 30 min of etching, the NWs reached a length of 8 μm with a highly compact morphology (Figure 2c), while increasing the etching time to 60 min resulted in disordered and irregular NWs with a length of 15 μm (Figure 2d). Based on these findings, a 30 min etching time was determined to be optimal and was chosen for subsequent experiments. These results align with Li and Duan^36^ findings, where they achieved SiNWs with a height of 9 μm after 30 min of etching using additional energy in the MaCE process with a solution of 5 M HF and 0.02 M AgNO_3_. By extending the etching duration to 1 h, they were able to increase the height of the SiNWs to 35 μm. Hoshian et al.^24^ obtain SiNWs with a height of 1 μm after 10 s of etching by using a 1:1 mixture of 50% HF and 30% H_2_O_2_ without adding IPA to the etchant solution.

**Figure 2:**
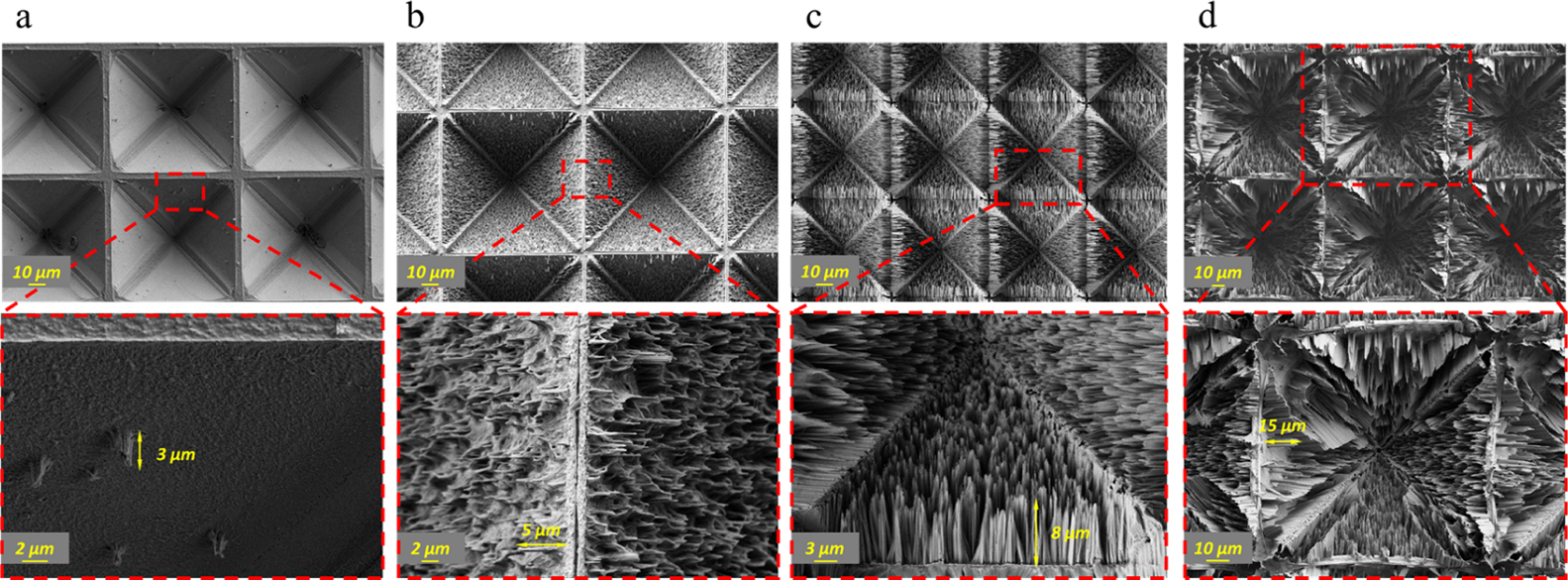
SEM micrographs show the effect of MaCE time on the length and density of SiNWs following etching for (a) 10 min, (b) 20 min, (c) 30 min, and (d) 60 min in HF: H_2_O_2_: IPA: DIW solution.

### 3.2 Hard Coating to Protect Nanowires

To determine the conversion factor of TiSi/Ti, 275 nm of titanium was sputtered on a planar silicon wafer and then annealed for 30 min and 2 h at 800 °C. The results indicated that after 30 min of annealing, the film thickness reached approximately 355 nm, and after 2 h, it increased to around 435 nm. This corresponds to the conversion factors of 1.3 and 1.6, respectively (Figure S8). The elemental composition of the hard-coated nanowires following silanization was examined using EDS (Figure S9). The EDS spectrum verifies that the nanowires were uniformly coated. Additionally, the uniform presence of fluorine on the surface is confirmed by both EDS and the surface’s wetting properties. The reaction between titanium and silicon in solid form occurs in three distinct stages. Initially, at lower temperatures, the diffusion of silicon leads to the formation of amorphous TiSi, resulting in an interlayer that grows as the temperature rises to 450 °C. During the second stage, polycrystalline silicides are formed in the temperature range of 450–650 °C. In the third stage, when the temperature exceeds 650 °C, the silicide transitions into C49–TiSi_2_, and at 800 °C, it further transforms into C54–TiSi_2_.^37,38^ Previous research has shown that annealing a 100 nm titanium film at 800 °C for 1 h results in the formation of a 251 nm TiSi layer.^38^ Similarly, a 60 nm titanium layer on a silicon substrate, under the same conditions, produces a 140 nm TiSi_2_ layer.^39^ However, in both studies, the samples were much thinner and not annealed with any additional gases, indicating that the film thicknesses, annealing atmosphere, and potential reactions could also impact the conversion factor.

XRD analysis confirmed the formation of TiSi and TiN after the annealing process. Figure 3a shows the XRD spectra for samples annealed in argon and nitrogen atmospheres. In argon atmosphere, peaks corresponding to C54 TiSi_2_ were observed at 30.10°, 39.25°, 42.35°, 44.12°, and 50.00°. When nitrogen was used as the annealing gas, in addition to TiSi formation, titanium reacted with nitrogen to form TiN. This reaction was confirmed by the appearance of a peak corresponding to TiN (111) at 2θ value of 36.82°.

**Figure 3:**
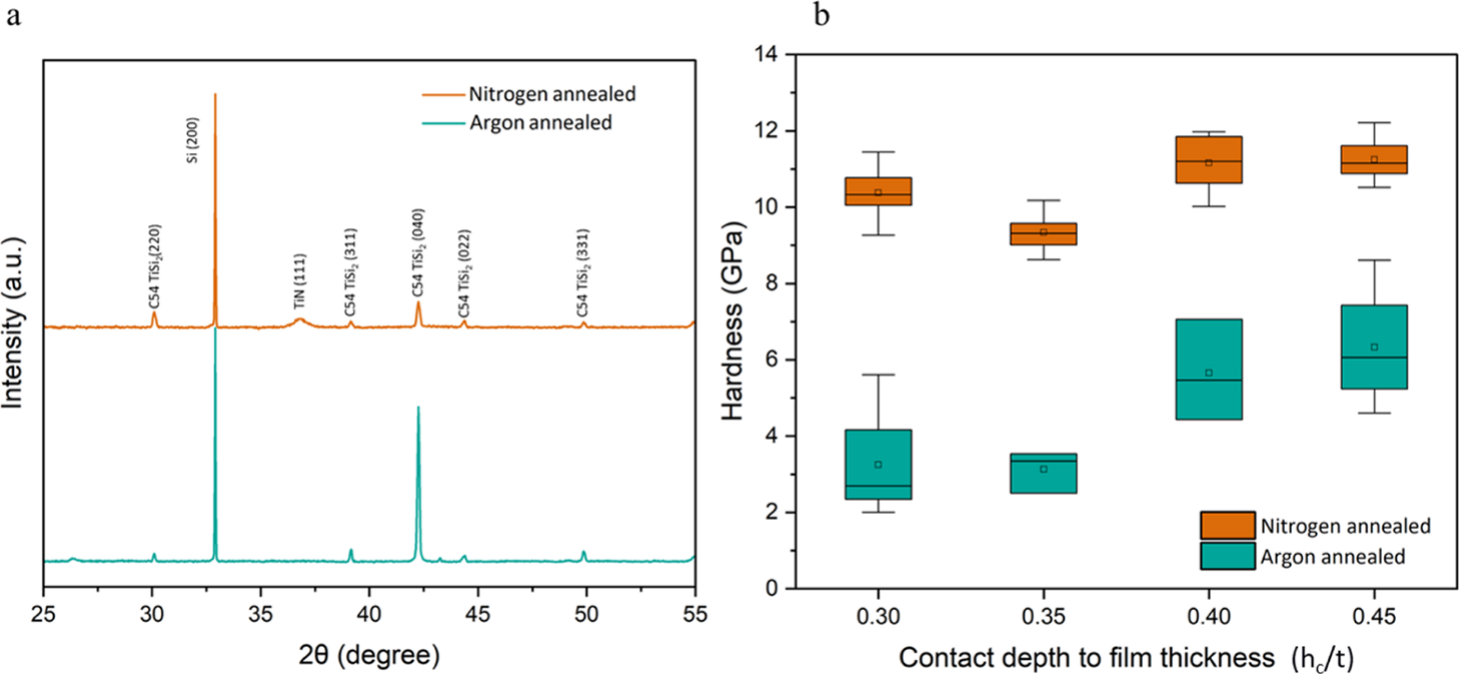
Characterization of hard coating: (a) XRD spectra of samples after 30 min annealing in argon and nitrogen atmosphere, and (b) variation of hardness as a function of contact depth to film thickness (*h*_c_/*t*) for samples subjected to the same annealing conditions.

Figure 3b presents the nanoindentation hardness measurements for surfaces annealed in argon and nitrogen atmospheres. It is evident that the hardness of the argon-annealed sample increases as the ratio of indentation contact depth to film thickness (*h*_c_/*t*) increases. This trend aligns with the expected substrate effect, where greater indentation depths result in increased influence from the underlying substrate. Previous studies have reported hardness values of 10–16 GPa for silicon substrates.^40,41^ Similarly, Vieira et al.^42^ observed hardness values of 3–4 GPa during nanoindentation of 3 μm titanium films deposited on polished 304 stainless steel substrates. The hardness results for the argon-annealed sample in this study closely match with those reported in the literature. For the nitrogen-annealed sample, the initial hardness values are lower but exhibit a slight increase at higher *h*_c_/*t* ratios. This behavior can be attributed to the formation and distribution of TiN and TiSi_2_ phases throughout the film volume. The presence of TiN notably enhances hardness, with values reaching approximately 11 GPa in the nitrogen-annealed sample. This observation is consistent with Fang et al.,^43^ who reported hardness values of around 10 GPa for TiN thin films deposited on silicon substrates via the plasma-enhanced chemical vapor deposition method, further supporting the formation of TiN under nitrogen-annealed conditions. Based on these results, samples annealed in nitrogen atmosphere for 30 min were used for the subsequent tests.

### 3.3 Ice Adhesion Strength

The ice adhesion test samples were prepared using SiNWs fabricated through 30 min of MaCE. Then, the samples were coated with titanium and annealed in a nitrogen atmosphere to enhance mechanical durability. For comparison, NW surfaces without hard coating were tested as reference samples. Figure 4a shows that at the first cycle, both surfaces have similar ice shear adhesion values (10 kPa) due to having similar morphology and surface chemistry. After 10 cycles, the same value is maintained for the two surfaces with and without hard coating. However, after this point, a clear difference in the ice shear adhesion values became apparent between the two surfaces, where the surface without a hard coating began to exhibit a significant increase in ice adhesion forces, reaching 22 kPa at cycle 20. On the other hand, the surface with a hard coating maintained a value of 10 kPa even after the 20 cycles. This suggests that the hard coating effectively protected the NW structures, preventing an increase in ice adhesion even after extended cycles.

**Figure 4:**
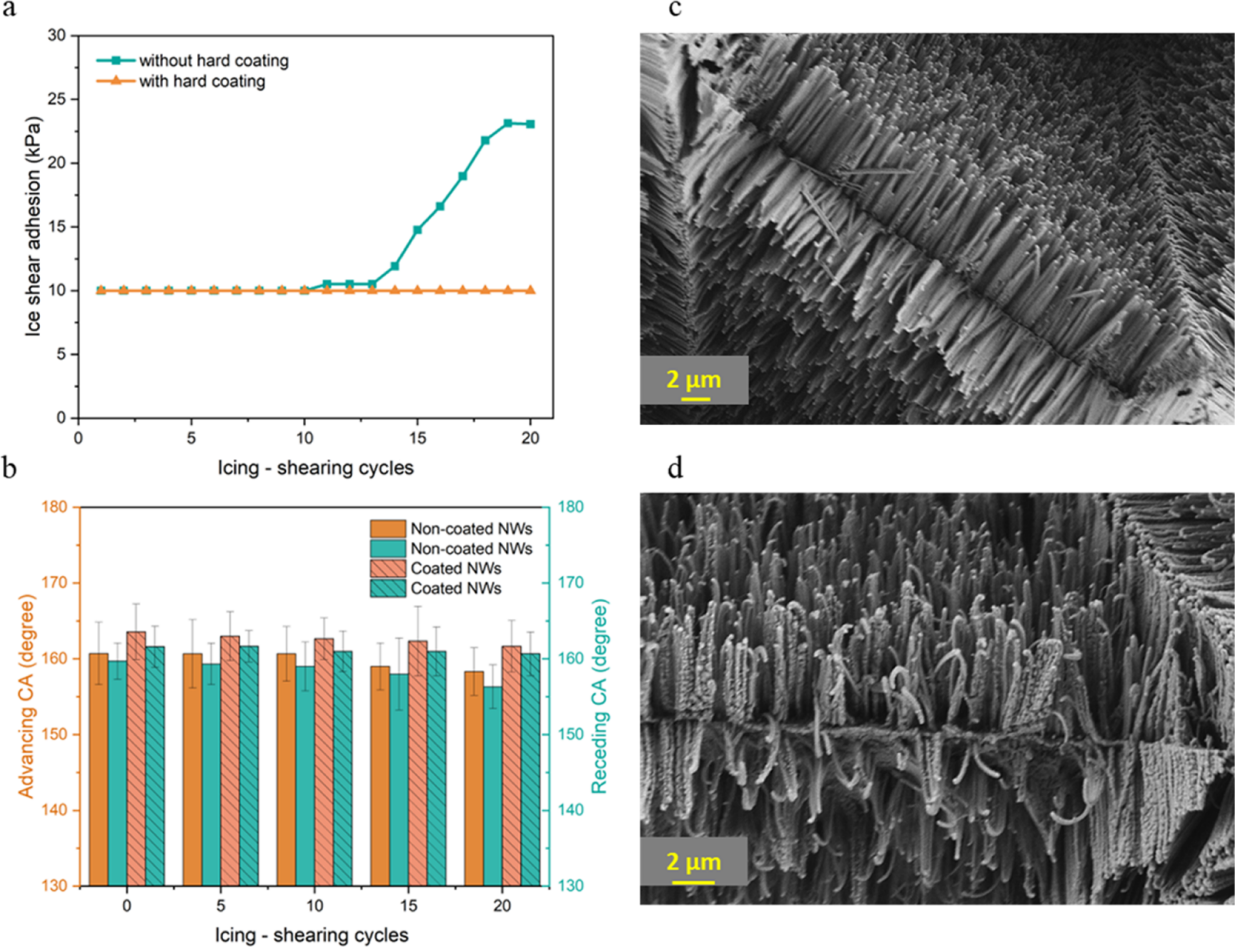
(a) Ice shear adhesion and (b) advancing and receding CAs of NW surfaces without and with hard coating over the 20 icing–shearing cycles (bars represent standard deviation). SEM micrographs of surfaces (c) with and (d) without hard coating after 15 icing shearing cycles.

The evaluation of surface wettability revealed that the hard-coated NW surfaces retained their superhydrophobicity, as both the advancing and receding angles remained mostly stable above 160° even after 20 ice-shearing cycles. Meanwhile, the noncoated surfaces experienced a modest decline, with both angles decreasing to roughly 155° (Figure 4b). Figure 4c shows that surfaces with hard coating experienced negligible wear, remaining visually unaffected by the ice-shearing tests. In contrast, noticeable deformation is evident on the NWs without a hard coating, as shown in Figure 4d. This deformation in NWs causes the breakdown of the Cassie state, enabling water to penetrate the nanostructure prior to freezing. This shift leads to a Wenzel-like state, characterized by increased contact between the ice and surface and enhanced mechanical interlocking, directly resulting in greater adhesion. The effectiveness of the hard coating is due to its ability to prevent this wetting transition caused by changes in surface topography. The ice detached from the NWs appeared less transparent compared to ice removed from a plain surface. This reduced transparency likely results from the formation of microcracks at the interface between the ice and the hierarchical surface structures, which may scatter light and create the observed translucency (Figure S10).

Researchers define icephobic surfaces as those exhibiting shear strengths ranging from 150 to 500 kPa, with some reports indicating values as low as 15.6 kPa.^44^ The ice adhesion strength below a threshold of 10 kPa is classified as a superlow ice adhesion surface.^45^ Chen et al.^46^ showed that the ice adhesion on SAM-coated NW structures applied to ablated microcones was initially extremely low, measuring around 2 kPa during the first few cycles. However, after 15 cycles, the adhesion increased to around 20 kPa and began to increase rapidly. Wang and Huang^47^ reported that water droplets exhibited lower ice adhesion strength (13.3 kPa at −20 °C) on a polypropylene replica with NWs. These NWs on replica surfaces help water droplets to remain suspended on the surface rather than allowing them to penetrate, which minimizes the solid–liquid contact area and reduces ice adhesion strength. Zhao et al.^48^ explored how ice adhesion strength varied on surfaces with regularly arranged pillars. They found that pillar spacings of less than 5 nm led to reduced ice adhesion compared to flat surfaces. Additionally, increasing the height of the pillars resulted in decreased ice adhesion strength, as more liquid water was confined within the textured surface.

### 3.4 The Durability Tests

To study both mechanical robustness and chemical stability of the resulting surfaces, the following tests were carried out: (1) sand impact, (2) sandpaper abrasion, and (3) immersion in acid, base, and solvent.

The sand impact and linear abrasion tests were conducted to evaluate the mechanical stability of both NW surfaces, both with and without hard coating. In the sand impact test, different sand weights were dropped from a height of 30 cm (Figure S11a). As shown in Figure 5a, the surfaces with hard coating retained high advancing and receding CAs even after being impacted by 60 g of sand, demonstrating their durability. In contrast, the surface without the hard coating experienced a remarkable decline in dynamic CAs, down to around 110° receding angle as the sand weight increased to 60 g, indicating a loss of superhydrophobicity. Calculating the CAH for both surfaces indicates that the hard-coated surface maintained a consistent CAH of approximately 5°, even with 60 g of sand, whereas the surface without a hard coating had a significant increase in CAH, reaching up to 25°. Regarding the SAs, the surfaces with hard coating still slid water droplets at around 20° even after being impinged by 60 g of sand, while the SAs of surfaces without hard coating exceeded 70° (Figure 5b). The surface morphologies of abraded samples revealed that the NWs on surfaces with hard coating experienced less damage after abrasion by 60 g of sand (Figure S11b) compared to samples without hard coating, where significant destruction of both micro- and nanostructures was observed (Figure S11c).

**Figure 5:**
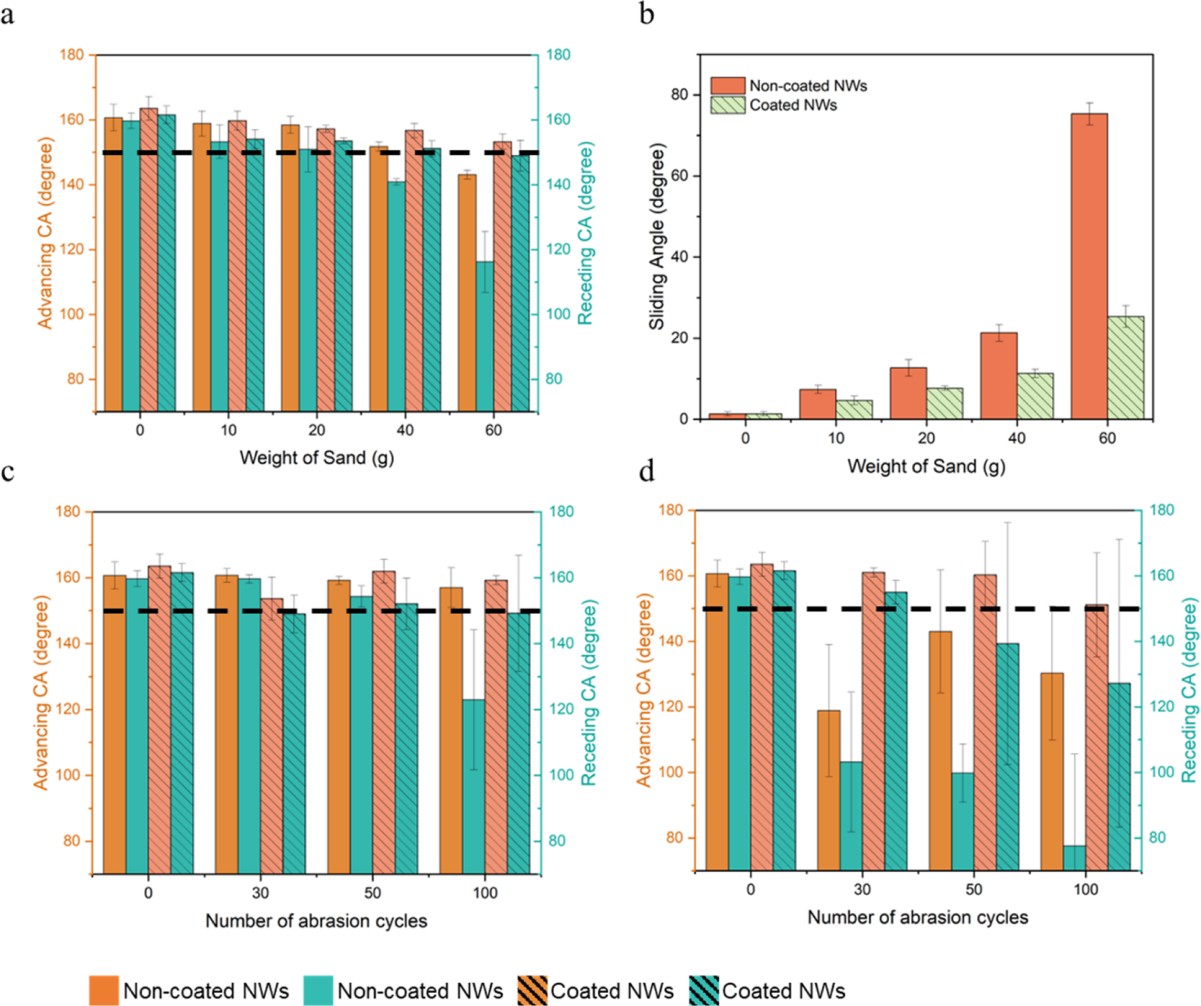
Wetting measurements: (a) the advancing and receding CAs, (b) SA values of surfaces without and with hard coating as a function of the weight of sand after being subjected to sand abrasion tests, and the advancing and receding CAs after being subjected to linear abrasion tests at different numbers of cycles using sandpapers (c) P4000, and (d) P2000. The black dashed lines serve as a visual aid for the eye, indicating a CA of 150° (bars represent standard deviation).

In the linear abrasion test, the findings revealed that hard-coated surfaces maintained their superhydrophobic properties more effectively than noncoated surfaces after 100 cycles of abrasion. With P4000 sandpaper, the advancing CAs on hard-coated surfaces remained consistent at roughly 160°, while receding CAs decreased by only 10°. In comparison, noncoated surfaces experienced a significant drop in receding CAs from approximately 160° to around 120° (Figure 5c). Using P2000 sandpaper, hard-coated surfaces exhibited advancing CAs that remained close to 150°, while receding CA values decreased to approximately 130°. Noncoated surfaces, however, experienced a notable decline in both advancing and receding CAs, dropping to 130° and 75°, respectively, after 100 cycles (Figure 5d).

This mechanical test demonstrated that the hard coating effectively protects the micro- and nanostructures, resulting in significantly enhanced mechanical robustness of the surface and greater durability of its superhydrophobicity. The surfaces with hard coating exhibited less damage to NWs compared to control samples, likely because of compressive residual stress. It was reported that high compressive residual stress values are generally induced in ceramic coatings prepared by physical vapor deposition due to the energetic ion bombardment during coating.^49^ Additionally, the collapsed hard-coated silicon nanowires may still contribute to the structure’s topography, unlike the uncoated NWs, which are destroyed by brittle fractures.

The nanowire-based structures were shown to significantly lose their ability to repel water when subjected to abrasion tests. This loss is due to the destruction of nanosized structures, which cannot maintain their rough topography during contact with sandpaper. However, the two-scale hierarchical structures fabricated using MaCE help prevent the complete destruction, allowing the surfaces to maintain their SHB properties with the CA of 155°.^23^ Hoshian et al.^24^ demonstrated that the nanostructures at the bottom remain intact even after abrasion, allowing a water droplet to partially penetrate the micropillars postabrasion, but it does not wet the bottom layer because the MaCE nanostructures beneath are still present.

Attempts to use metal silicide or nitride to mechanically protect surface structures in the literature have been very limited. Transition metal nitride coatings are utilized to preserve micro- and nanostructures due to their hardness, resistance to fatigue, excellent corrosion resistance, and chemical stability. For instance, TiN shows remarkable mechanical properties, making it a proper candidate for wear-resistant coatings.^50^ Gao et al.^51^ showed that TiN coatings deposited through multiarc ion plating were anodized to create composite structures containing embedded TiO_2_ nanotubes, followed by a hydrophobic treatment. These TiN hard-coated surfaces exhibited antiwetting characteristics after undergoing 20 cycles of linear abrasion and 100 cycles of tape peel tests. DeFlorio et al.^52^ reported that the static CA of a SHB nanodiamond-based coating decreased from approximately 155° to around 140° and 135° after undergoing 50 and 100 cycles of abrasion with sand particles measuring 300 μm in diameter.

In terms of chemical stability, the hard-coated surface retains its superhydrophobic properties even after being submerged in different harsh solutions for 6 h. The CA measurements showed that both advancing and receding angles stayed nearly unchanged, with minimal and consistent hysteresis, enabling water droplets to remain in the Cassie–Baxter state (Figure S12).

### 3.5 Plastron Stability

To further evaluate the impact of sand abrasion on the durability of the surface’s superhydrophobicity, air plastron stability in water was measured for both intact hard-coated and noncoated surfaces, along with their 10 g sand-abraded counterpart. Figure 6a presents the plastron coverage ratio over time upon water immersion. The air plastron of both nonabraded surfaces (with and without hard coating) exhibited significant stability (Figure S13a), lasting over a month with minimal decay. This stability arises from the integration of highly rough hierarchical structures formed by silicon wet etching, which creates microstructures, and MaCE methods, which produce nanostructures. This is further reinforced by the application of a hydrophobic coating, which significantly contributes to maintaining the stability. The abraded SiNWs with hard coating maintained a relatively long microplastron stability, highlighting their superior mechanical resistance to sand abrasion. In contrast, the SiNWs without hard coating showed an immediate 10% drop in plastron coverage ratio compared to their nonabraded counterparts, primarily due to the extensive damage caused by sand abrasion. After 60 h of immersion, the abraded sample with hard coating showed only around 20% microplastron coverage loss. On the other hand, the abraded sample without the hard coating experienced a 2-fold reduction in plastron coverage compared to its nonabraded reference (Figures 6b, S13b). This highlighted the protective role of hard coatings that maintained the nanoscale structures, ensuring the surface retained the high capillary pressure necessary to keep water in the Cassie state, effectively preserving the nanoplastron. Tesler et al.^29^ also observed plastron loss on a Ti-coated SHB surface due to sand abrasion.

**Figure 6:**
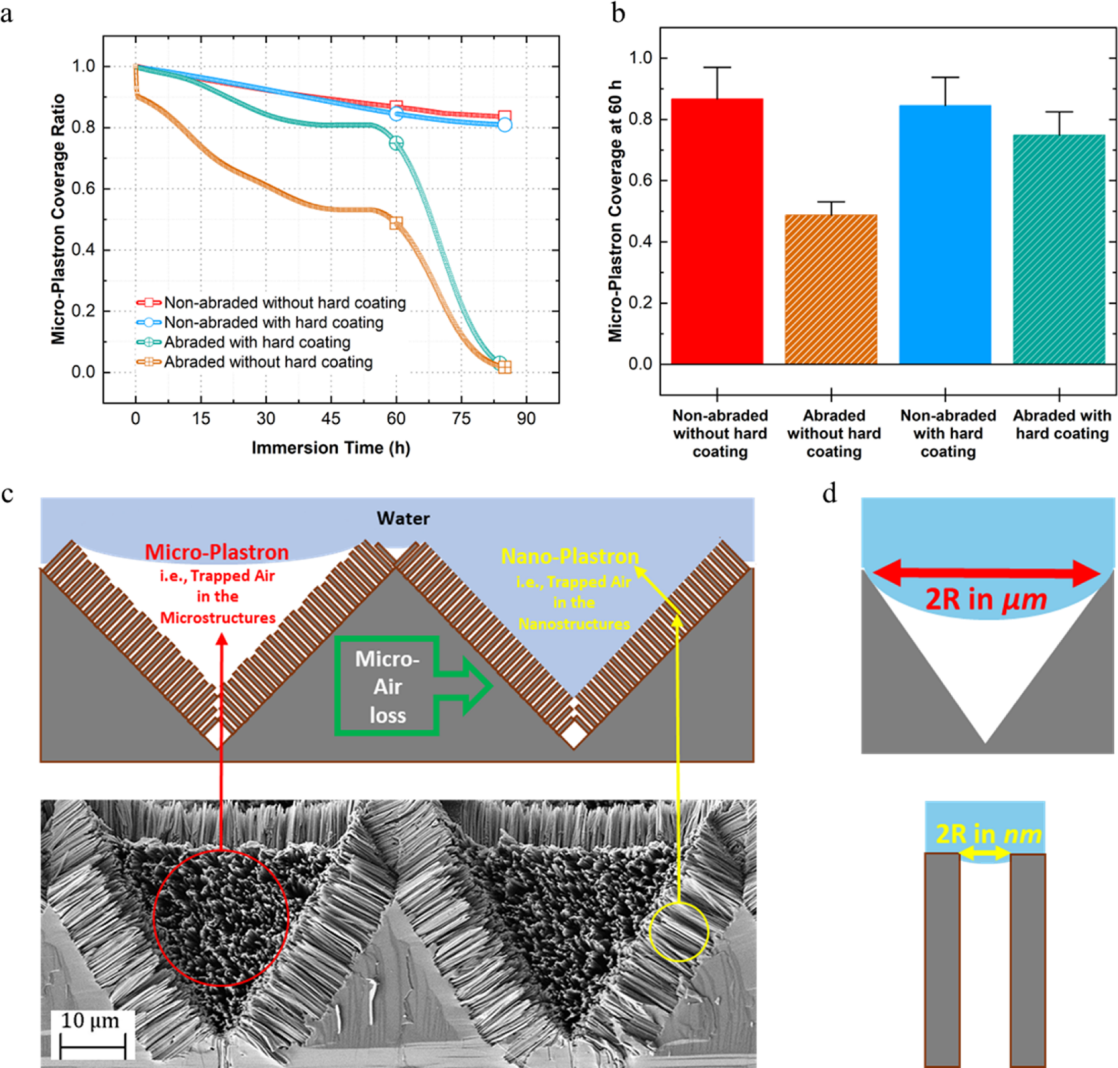
Effect of sand abrasion (10 g) on the plastron stability of NWs with and without hard coating. (a) Microplastron coverage ratio over immersion time in water for abraded and nonabraded samples of each NW surface. (b) A comparison of the microplastron coverage ratio of the four samples after 60 h of immersion. (c) A schematic illustration (top) of the concepts of micro- and nanoplastron and SEM image (bottom) of the NW surface for demonstration. The loss of the trapped air (green box with arrow) in the microstructures will drive the formation of a nanoplastron. (d) Schematic illustration of a droplet curvature of an inverted pyramid (up) and between adjacent NWs (bottom). *R* is the curvature tip radius.

Figure 6c demonstrates the concepts of micro- and nanoplastron. The microplastron (i.e., air trapped in the microstructures) of both abraded surfaces is lost after around 85 h. The inverted pyramid microstructures, with their periodic architecture, form a continuous air plastron film, which is vulnerable to surface defects. As demonstrated by Awashra et al.,^33^ mechanical defects, such as missing structures on SHB surfaces with periodic and open micromorphology, can critically compromise plastron stability. Nonetheless, the nanoplastron (i.e., contained within the NWs) is significantly stable and survives for more than several months. According to the Young–Laplace eq (eq 1), the very small spacing between the adjacent NWs (2R) significantly increases the liquid–air interface stability and the critical pressure value needed to overcome its capillary forces (*P*_c_),^53^ as shown in Figure 6d.

1 $$P_{c}=-\frac{2\gamma_{lv}\mathrm{COS}\left(\theta \right)}{R}$$

where γ_lv_ is the liquid–vapor surface tension, and θ is liquid CA on the planar surface. Poetes et al.^54^ also observed that the liquid–air interface gradually moved down the surface structures, shifting from the microstructures to the nanostructures, leading to a decrease in air plastron film thickness.

## 4 Conclusion

We successfully fabricated SiNWs within pyramidal microstructures using the MaCE method to achieve dual micro- and nanohierarchical structures. Titanium was deposited on the NWs by sputtering, followed by annealing in a nitrogen atmosphere, leading to the formation of TiSi and TiN, as confirmed by XRD analysis. This hard coating enhanced the mechanical durability of the surface by protecting the hierarchical structures from mechanical wear. SEM analysis showed that the hard-coated samples had fewer damaged pillars compared to controls. Furthermore, it could be that the collapsed hard-coated SiNWs are still able to contribute to the structure’s topography, unlike the SiNWs without hard coating, which are destroyed by brittle fracture. The surfaces with hard coating demonstrated increased durability of SHB properties, including low SAs and hysteresis, along with low ice adhesion, strong resistance to mechanical stress, such as sand impact, and extended air plastron lifetime. Therefore, the application of a hard coating to protect nanostructures could be beneficial for any application where proper function depends on fragile nanostructures.

## Supplementary Material



## Notes

Additional SEM images, EDS analysis and respective element mapping, supplementary experimental details including photographs of devices, experimental setup and samples, contact angle measurement chart after chemical stability test, and table of annealing steps (PDF)
