# Supplementary material for: Durable Icephobic and Superhydrophobic Silicon Nanowire Surfaces
Source: ACS Appl Mater Interfaces. 2025 Nov 17;17(47):65242–52. doi: 10.1021/acsami.5c13616 (PMC12673527; doi:10.1021/acsami.5c13616)
Supplement: Supplementary file 1 [file am5c13616_si_001.pdf]

Supporting Information

Durable Icephobic and Superhydrophobic Silicon Nanowire Surfaces

Seyed Mehran Mirmohammadi [a*], Miika Heikkilä [a], Laura Fieber [a,b], Mohammad Awashra [a], Sara Hamed [a], Suprit Bhusare [c], Gaurav Mohanty [c], Robin H. A. Ras [b,d], Ville Jokinen [a], Sami Franssila [a*]

[a] *Department of Chemistry and Materials Science, Micronova Nanofabrication Centre, Aalto University, Espoo, Finland.*

[b] *Department of Applied Physics, Aalto University, Espoo, Finland.*

[c] *Faculty of Engineering and Natural Sciences, Hervanta Campus, Tampere University, Tampere, Finland*

[d] *Centre of Excellence in Life-Inspired Hybrid Materials (LIBER), Aalto University, Espoo, Finland.*

Seyed Mehran Mirmohammadi – Department of Chemistry and Materials Science, Micronova Nanofabrication Centre, Aalto University, Espoo, 02150, Finland; Email: mehran.mirmohammadi@aalto.fi

Sami Franssila – Department of Chemistry and Materials Science, Micronova Nanofabrication Centre, Aalto University, Espoo, 02150, Finland; Email: sami.franssila@aalto.fi

1. Supplementary Figures

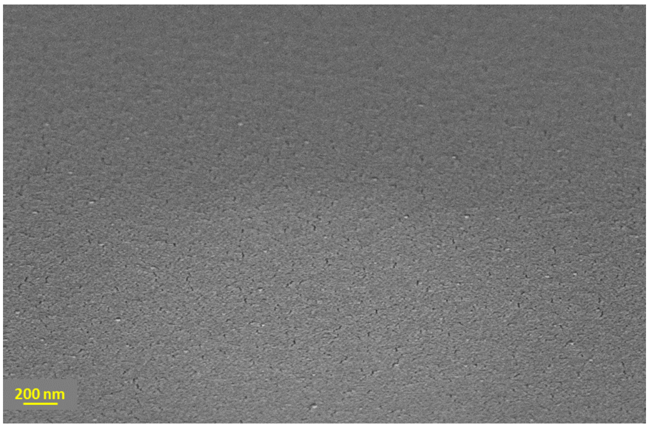

Figure S1. The SEM images of 10 nm sputtered gold on a plain silicon wafer.

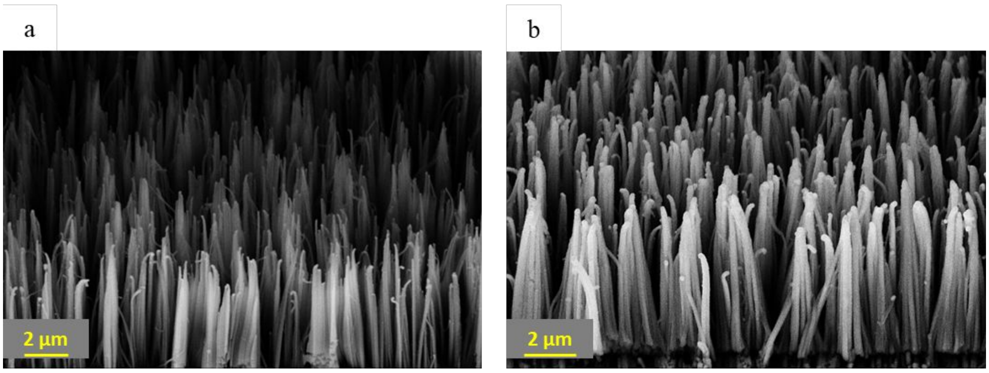

Figure S2. SEM micrographs show NWs before and b) after titanium annealing in a nitrogen atmosphere at 800 ˚C for 30 min.

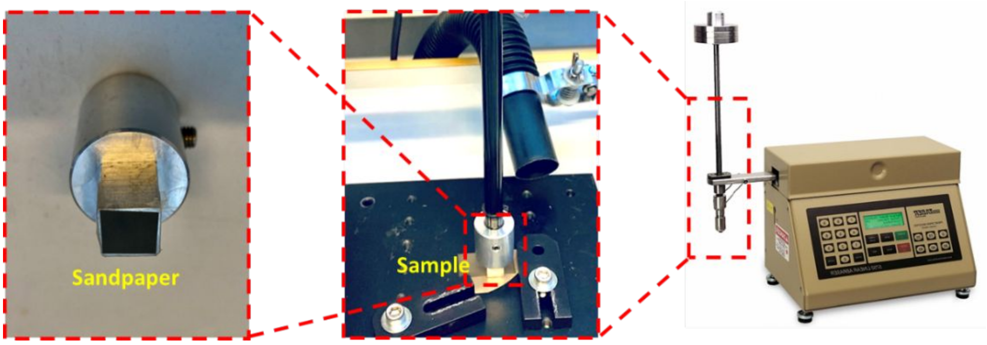

Figure S3. The Taber linear abrader device for the abrasion test secures the sample in the holder along with the sandpaper.

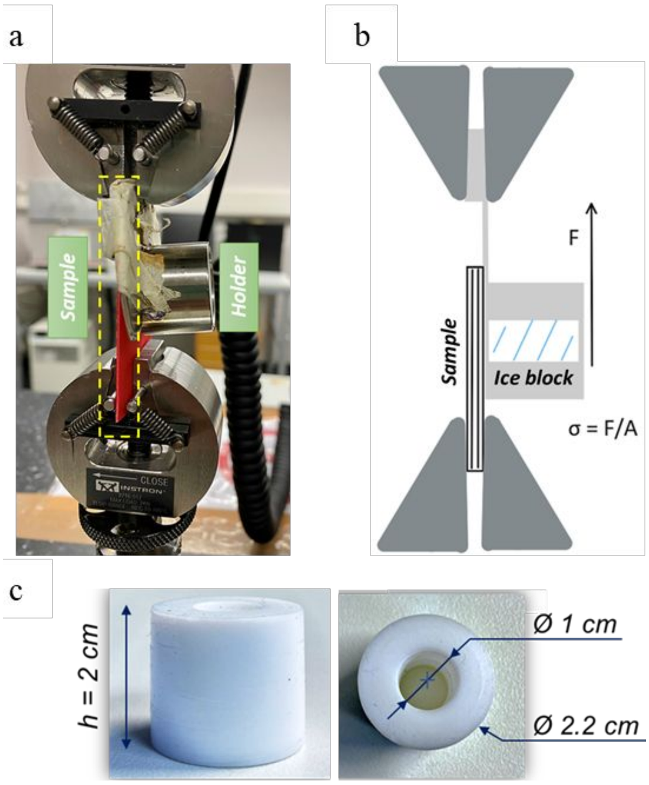

Figure S4. a) The Instron 4204 device for the ice adhesion pull-off test. b) A schematic illustrates the positions of the ice block and test sample. c) A cylindrical Teflon mold, measuring 1 cm in inner diameter, 2.2 cm in outer diameter, and 2 cm in height, was used to prepare the ice blocks.

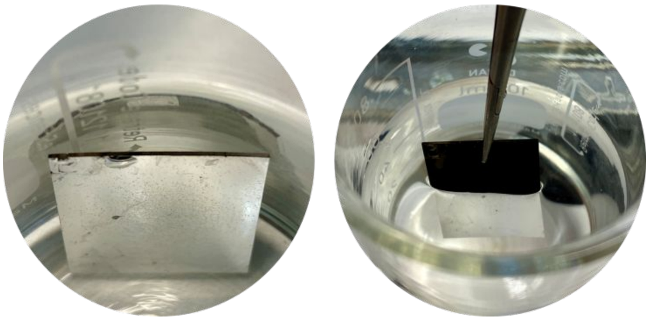

Figure S5. Photos of silicon nanowires with the hard coating, which have been submerged in water for several months, reveal a mirror-like surface.

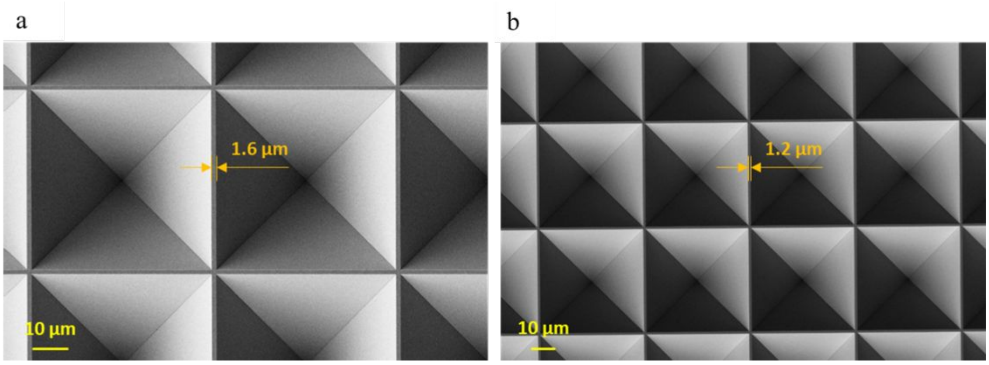

Figure S6. The SEM images of a) inverted pyramidal structures after 25 min, and b) 30 min etching in KOH solution at 80 ˚C.

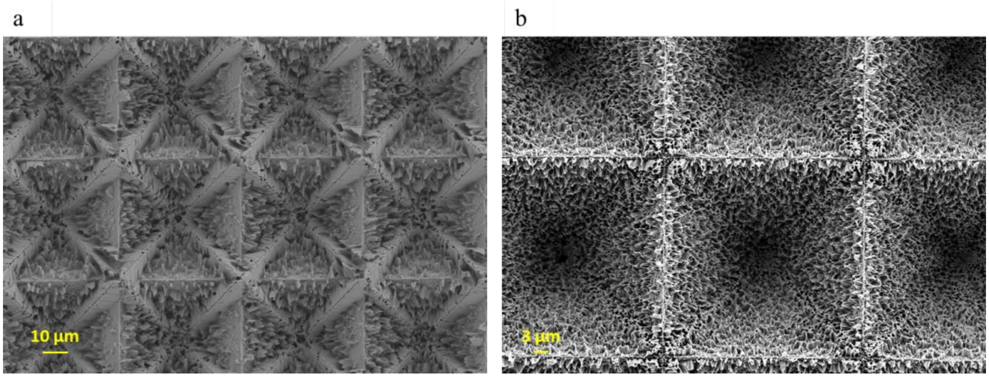

Figure S7. The SEM images of NWs formation in which no IPA was added to the etchant solution: a) without and b) with stirring during the MaCE process.

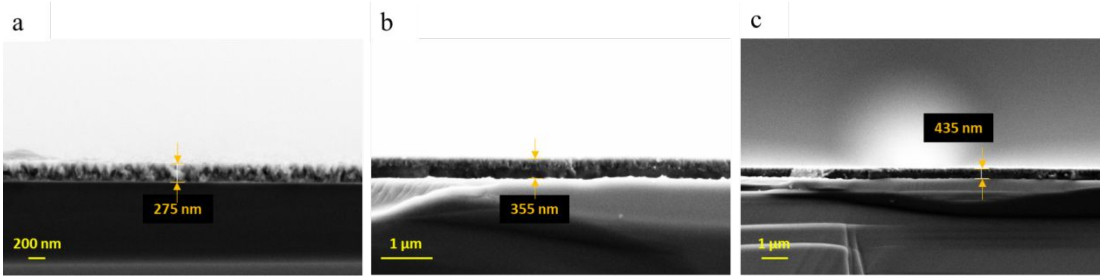

Figure S8. The cross-section SEM images of a) sputter titanium, b) film layer after 30 min, and c) 2 h of annealing at 800 ˚C on planar silicon wafers.

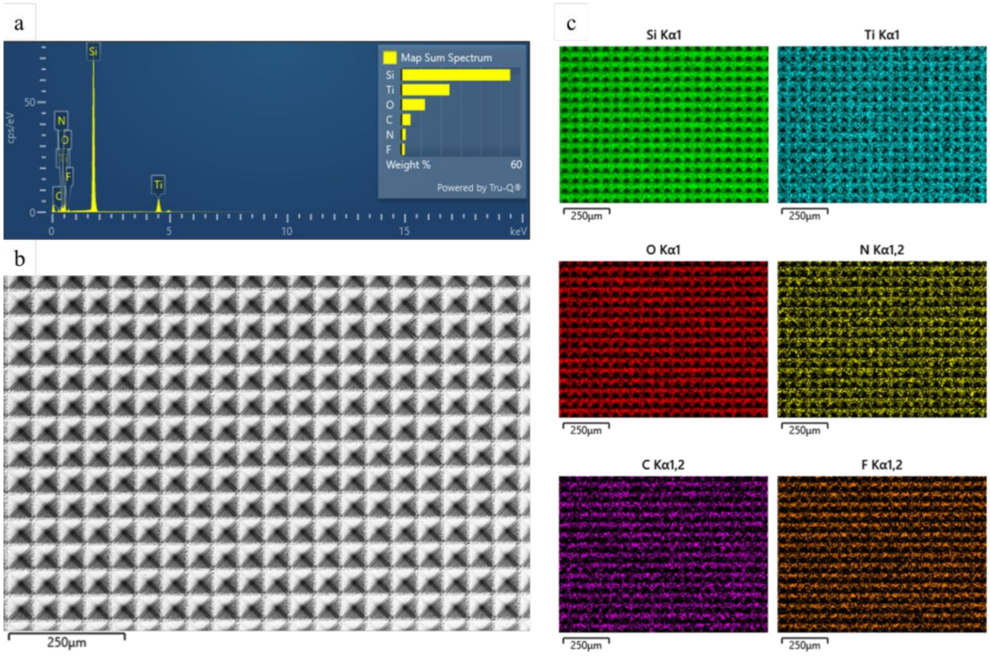

Figure S9. EDS analysis of resulting surface: (a) the energy spectrum covering all elements, (b) The SEM image of hard-coated NWs after silanization, and (c) respective element mapping.

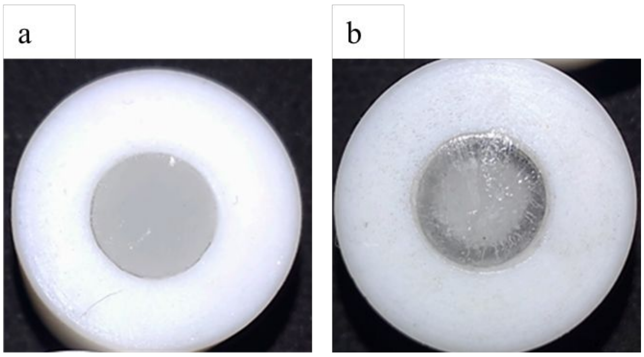

Figure S10. Ice detachment from a) NWs and b) plain polydimethylsiloxane (PDMS) surfaces.

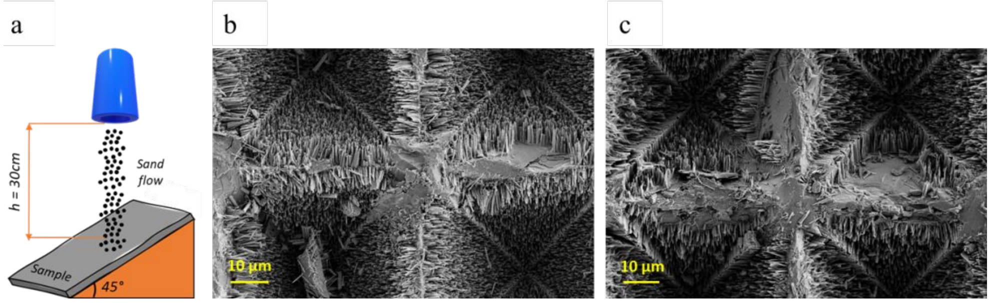

Figure S11. a) Schematic of sand impact test in which sand was released from a height of 30 cm. The SEM images of b) hard-coated and c) non-coated NWs after being impinged by 60 g of sand.

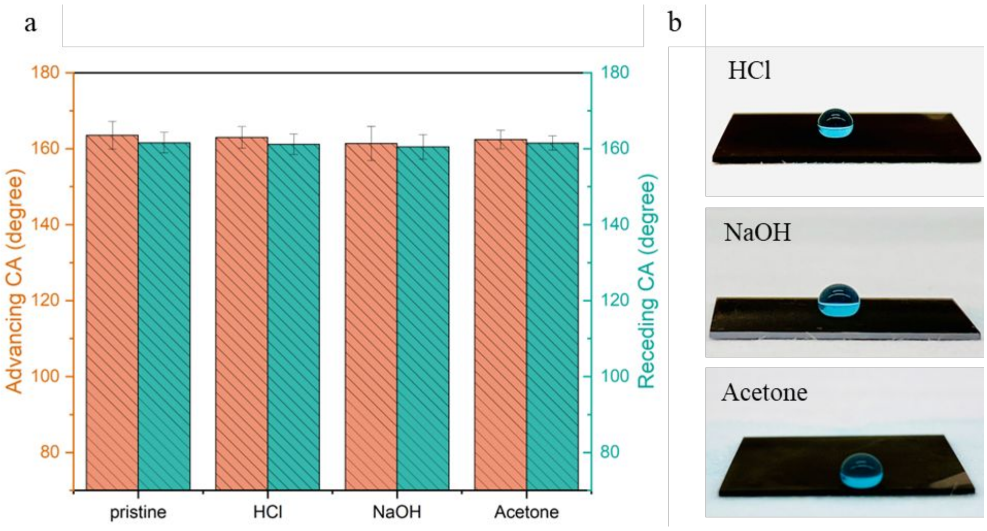

Figure S12. a) The advancing and receding CAs of hard-coated surfaces after 6 hours of immersion in different solutions (bars represent standard deviation), and b) photos of colored water droplets on hard-coated surfaces after being immersed for 6 hours.

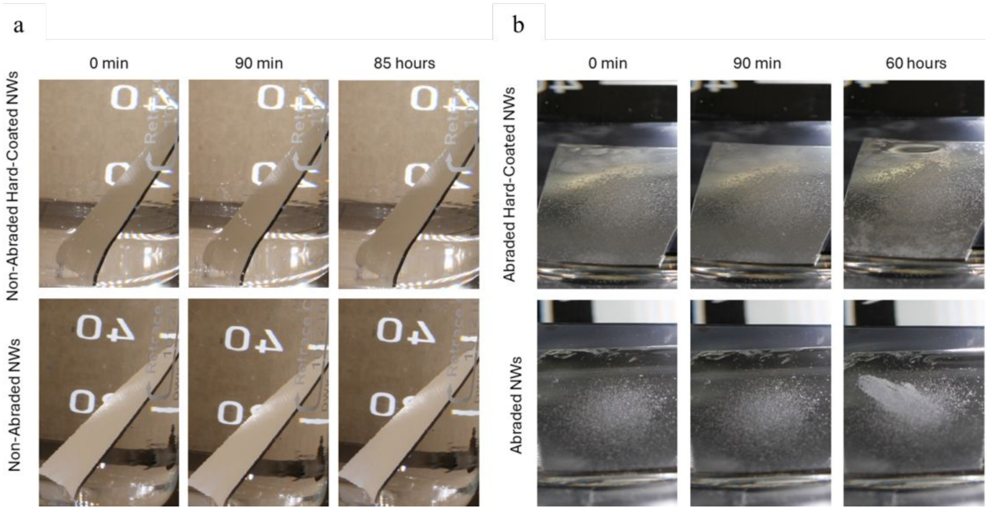

Figure S13. Photos of hard and non-coated surfaces completely submerged in water at different stages: a) non-abraded surfaces, and b) sand-abraded surfaces.

2. Supplementary Table

Table S1. The condition of the titanium annealing steps at a vacuum of 28 mbar.

step	time (min)	temperature (˚C)	slop (˚C/min)	gas flow (slm)
1	15	350	21.9	3
2	25	500	6.0	3
3	20	750	12.5	3
4	5	800	10.0	3
5	30	800	0.0	3
6	12	500	-25.0	3
7	10	350	-15.0	3
8	17	200	-8.8	3
9	15	100	-6.7	3
10	8	50	-6.3	3
